# Shock after Operation

**Published:** 1914-04-15

**Authors:** 


### SHOCK AFTER OPERATION.

Physicians, surgeons, anesthetists and laboratory scientists are not agreed as to the best treatment for this condition, or whether there is any best treatment. Dr. S. T. Pope[1] of San Francisco has recently presented a paper describing his experimental study of shock in animals. The results of various treatments of shock induced in animals by bleeding are graphically demonstrated by blood-pressure tracings. The usefulness of many drugs used in shock is apparently proved from the experimental point of view.

The clinical objection to drawing positive conclusions from laboratory findings and applying such conclusions practically in the operating-room is that a patient suffering from shock has rarely lost much blood. Also, the patient has generally been subjected to the nervous strain of apprehension for some time before the operation is performed. There has also been a more or less prolonged abstinence from food, depending on the rule of procedure of the operator or the anesthetist. The method of producing anesthesia is also important; that is, whether or not preinjections of

1 Pope, S. T.: California State Jour. Med. December, 1913. p. 499.

morphin, scopolamin or other alkaloids have been made, or whether or not Crile's method of preventing shock has been followed. The cause of shock in a person operated on is very different from that in an animal, which, by bleeding, or other measures, is deliberately shocked. Consequently, successful treatment not only may be, but perhaps must be, different.

Many incontrovertible truths are offered by Dr. Pope. There is no question that when drugs are administered hypodermatically, it must take a considerable time in minutes (the length depending on the drug used) before any results can be expected in improved cardiac strength or blood-pressure. In the next place, it is not always justifiable to conclude that a drug administered hypodermatically has exactly the same action as when administered intravenously. The intensity of the intravenous action is very different from the slowly absorbed or possibly local (irritant?) action of a drug administered hypodermatically.

While camphor is shown by Pope to be of no value in shock when given to animals intravenously and to cause an actual fall in blood-pressure, when administered in the usual manner, subcutaneously, the circulation is certainly improved by its use, although, in discussion, he stated that this improvement may be only apparent, in that the amplitude of the pulse is increased, although the blood-pressure may not be. In any case, when a pulse is almost absent at the wrist and improves under the action of camphor subcutaneously, and the color returns to the patient's face, we must believe that the camphor has caused some improvement of the person in shock. Also, it is a fact that there is no after-depression following such camphor injections. The same improvement was often found when, a few years ago, brandy or whisky was administered subcutaneously, or when, as often in Germany in previous years, ether was so given. From reflex irritation, before much was absorbed, the circulation certainly improved. Later, after absorption, however, the depression increased. That is, the future of alcohol as a stimulant is bad, while the future of camphor in ordinary dosage is apparently good.

Pope shows what the profession should learn more rapidly, that, in serious conditions, strychnin does not raise the blood-pressure, and may do harm to a wornout, fluttering, rapid heart. On the other hand, in prolonged shock, when the nerve-centers appear to be sluggish, the impulses apparently lack tone, and the patient seems to be slowly dying with cerebral and nervous apathy, strychnin is certainly valuable, in not too large doses.

The sudden dilatation caused by nitroglycerin seems pharmacologically entirely incorrect in shock. The increase in peripheral circulation which it causes can better be secured by the use of dry heat and perhaps general massage or friction.

Pope does not mention atropin. This alkaloid is very slow in its action, but it is a respiratory and cardiac stimulant and generally improves the arterial pressure. Therefore, whatever immediate treatment of a patient is used, a sufficient dose of atropin may well be given hypodermatically to act in a few hours, if other measures save the patient from immediate death.

Some physicians have occasionally employed cocain in shock. Because of its pharmacologic activities, its use appears inexcusable.

Digitalis in any form does not seem indicated in shock, but strophanthin, given intravenously, has been repeatedly shown (and again by Pope) to cause an improved heart-action and increased blood-pressure. This drug does not act so well when given subcutaneously or intramuscularly.

As we would expect, injection of epinephrin is shown by Pope to cause an enormous rise in blood-pressure. He does not tell us how long this rise was sustained, but its action has been found so evanescent that few clinicians or surgions now rely on it. To be of value, a minute dose must be constantly administered, intravenously, in physiologic saline or in Ringer's solution. At times it has seemed that subcutaneous injections of epinephrin solution were valuable for more or less continuous improvement, this probably because such local contraction is caused that but a little is absorbed at a time; so that such an injection may give continuous epinephrin treatment for some time. On the other hand, there is danger of abscesses from such disturbance of the tissue as epinephrin can cause.

Pope did not experiment with the pituitary blood-pressure-raising agent, but such extracts seem to be more valuable than epinephrin in conditions of shock. The blood-pressure rise is not so great, but is more prolonged and the action on the medullary centers is more satisfactory, as epinephrin may cause respiratory depression. Also, pituitary extracts are more or less diuretic.

Caffein, given intravenously, is shown by Pope not to raise the blood-pressure, but apparently even to lower it. It is a question whether caffein, given by mouth or rectum or hypodermatically, would ever reduce the blood-pressure, even if it did not raise it immediately. There can be no disputing the fact that caffein can cause a great deal of irritability, palpitation and weakening of the force of the heart. Also, some patients show a very disturbing idiosyncrasy to caffein. Generally, however, in depressed circulatory conditions, as in mild shock or when disturbed circulation occurs in serious illness, caffein is one of the best circulatory stimulants that we possess.

Pope well urges that when rapid breathing has occurred prior to or during an operation, and the patient is in a condition of acapnia, and the blood-pressure has fallen from loss of carbon dioxid, as well demonstrated by Henderson, the patient really is suffering also from oxygen starvation. Such a patient needs oxygen, perhaps administered mixed with carbon dioxid; but artificial respiration, or respiration by means of a pulmotor, or the administration of small doses of oxygen (not to hyperoxygenate the patient), or the administration of oxygen mixed with carbon dioxid, are positively essential.

Pope urges that with the respiration and circulation improving through the oxygen inhalations, the carbon dioxid will again be formed in the tissues and the patient will develop the carbon dioxid stimulation of the blood-vessels and the respiratory center. In this way he interprets Henderson's findings for practical application in the operating-room. He does not speak of inverting the patient and thus increasing the blood-pressure at the base of the brain. Such simple physical therapy should never be forgotten.

On the other hand, a prolonged Trendelenburg position can cause disturbed heart-action, or even a heart-block, Pope says, "due either to acute dilatation, to toxins, or fatigue, or sepsis, or coronary clots, or direct trauma to some area of cardiac reflex." It is quite possible, however, that from this position slight edema or exudate into the fourth ventricle may occur and may disturb the respiratory and pneumogastric centers. In shock from this cause, Pope suggests venesection from the jugular vein, cardiac massage and epinephrin in Ringer's solution forced backward in the arterial stream, as giving the only possible chance for recovery.

If respiration is stopped from obstruction, an entirely different condition arises. All that is needed, when this occurs, is to lift the jaw, pull forward the tongue, clean out the mouth and larynx, and use artificial respiration or a pulmotor.

A good immediate treatment of shock, until a better is offered, would be to start artificial respiration or respiration by means of the pulmotor at once; to raise the feet and legs if the patient has not been in a Trendelenburg position; to give one or more hypodermics of camphor (with all due respect to the objection of Dr. Pope); to administer atropin sulphate 1-100 grain for its future action (provided atropin in some form has not already been administered as a prelude to the anesthesia); to give strophanthus intravenously if deemed advisable; or to give a hypodermatic injection of pituitary extract, unless it shall be shown to be of no great value. The patient should be surrounded with hot-water bags; friction of the extremities is advantageous; coffee may be administered by rectum, unless it is considered of advantage to give caffein hypodermatically, this also for its future action if the patient quickly improves from the activities of the more immediately acting drugs.—*Journal A. M. A.*, March 7, 1914.

